# A Case of Invasive *Aspergillus* Rhinosinusitis Presenting with Unilateral Visual Loss and Subsequently Associated with Meningitis, Subarachnoid Hemorrhage, and Cerebral Infarction

**DOI:** 10.1155/2020/8885166

**Published:** 2020-09-08

**Authors:** Koji Tsuzaki, Kenji Murakata, Mayu Kamei, Akihiro Kikuya, Yuwa Oka, Toshiaki Hamano

**Affiliations:** ^1^Department of Neurology, Kansai Electric Power Hospital, Osaka, Japan; ^2^Division of Clinical Neurology, Kansai Electric Power Medical Research Institute, Osaka, Japan; ^3^Department of Neurology, Nakatsu Hospital, Osaka, Japan

## Abstract

Visual impairment can occur because of several mechanisms, including optic nerve disease and occasionally fungal sinusitis. An 87-year-old man presented with the loss of right visual acuity; he was diagnosed with optic neuritis. Steroid pulse therapy was not effective. One month later, he became unconscious because of meningitis, following which treatment with ceftriaxone and acyclovir was initiated. However, his consciousness deteriorated because of a subarachnoid hemorrhage caused by a ruptured aneurysm. Meningitis and vascular invasion caused by fungal rhinosinusitis were suspected, and the sinus mucosa was biopsied. He was pathologically diagnosed with invasive *Aspergillus* rhinosinusitis. Despite continuous liposomal amphotericin B administration, he died of cerebral infarction, following a right internal carotid artery occlusion. It is important to consider the possibility of *Aspergillus* as an etiological agent, especially when cerebrovascular events are associated with visual impairment.

## 1. Introduction

Unilateral visual loss is commonly caused by problems of the optic media, retina, or neural visual pathway. The optic nerve is included in the neural visual pathway. Causes of optic neuropathy include ischemia, inflammatory, or infectious process. When optic neuritis is diagnosed, it is commonly treated with steroid pulse therapy [1]. However, it should be noted that fungal rhinosinusitis (FRS), such as *Aspergillus* rhinosinusitis, infrequently causes unilateral visual loss.

The presentation of FRS could vary from being irritating to potentially fatal [2]. FRS is categorized as invasive because the fungus invades the surrounding tissues, such as bone, or maybe noninvasive [2]. Invasive FRS cases are subdivided into acute, granulomatous, and chronic [2]. In acute invasive FRS, vascular invasion rapidly progresses within 4 weeks [2]. Acute invasive FRS, though rare, has a 50–80% mortality rate [3]. An immunosuppressed status, including neutrophil dysfunction, chemotherapy, and human immunodeficiency virus infections, is the risk factor for FRS [4]. In this report, we describe a case of invasive *Aspergillus* rhinosinusitis that presented with unilateral vision loss, meningitis, subarachnoid hemorrhage, and cerebral infarction.

## 2. Case Presentation

**Figure 1:**
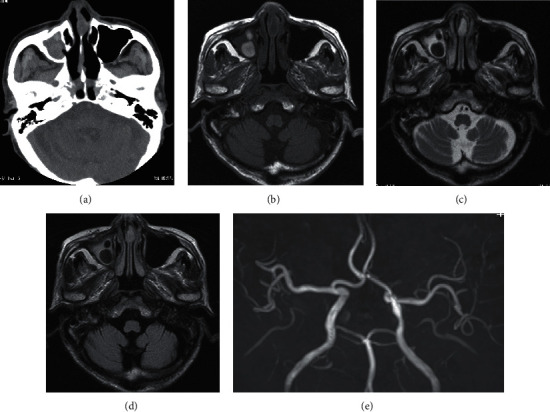
One month before admission: imaging included CT (a), T1-(b), and T2-weighted (c) images, fluid attenuated inversion recovery images (d), and MRA (e).

An 87-year-old man presented with vision loss of the right side one and a half months before admission. He had consulted an ophthalmologist one month before admission. There was no abnormality in the fundus, and computed tomography (CT) of the head revealed no abnormal findings, except sinusitis on the right side (Figure 1(a)). On brain magnetic resonance imaging (MRI), mass lesions in the right maxillary sinus were seen with a high signal intensity on T1-weighted images and with a low signal intensity on T2-weighted and fluid-attenuated inversion recovery images (Figures 1(b)–1(d)). No aneurysm was found on magnetic resonance angiography (MRA) (Figure 1(e)). He was subsequently diagnosed with optic neuritis and treated with steroid pulse therapy. However, his visual acuity did not improve, and hearing disturbance developed in the right ear. In the evening on the day of admission, he became unresponsive, and abnormal behavior, such as wandering, was observed. He was brought to our hospital by ambulance. He had a medical history of hypertension and benign prostatic hyperplasia. He had not taken steroids or immunosuppressants.

At the time of admission, the patient's Glasgow coma scale was E4V4M6, and he could spontaneously open eyes. He was disorientated and could follow only simple instructions. Both light blink and direct light reflexes disappeared in the right eye, and a relative afferent pupillary defect was observed in the right eye. His eye position was midline. Although he could not move his eyes as instructed, he spontaneously moved them in all directions. Visual acuity could not be assessed because of disturbance of consciousness. There were no abnormalities in motor and sensory examinations. His deep tendon reflexes were normal, and signs of meningeal irritation were absent.

**Figure 2:**
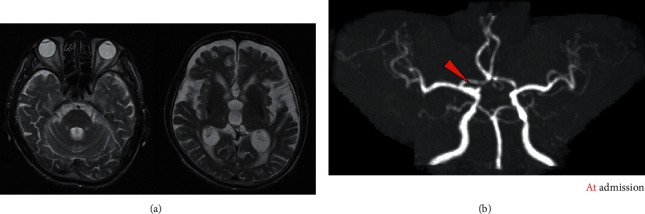
On admission: brain MRI showing no abnormalities in the cerebraand parenchyma on T2-weighted images (a). MRI shows a 3 mm aneurysm in the siphon of the internal carotid (arrow head) (b).

On laboratory examination results, his white blood cell count was mildly elevated (10700 cells/*μ*L; normal range, 3500–9000), and C-reactive protein level was elevated (4.68 mg/dL; normal range, 0–0.3). *β*-D glucan level was normal (3.259 pg/mL; normal range, <11.0; colorimetric assay). Tests for the presence of autoantibodies, including antinuclear antibodies and antineutrophil cytoplasmic antibodies, were negative. Cerebrospinal fluid (CSF) examination findings showed slightly elevated cell counts (65 cells/*μ*L; mononuclear cells, 50 cells/*μ*L; polymorphonuclear cells, 15 cells/*μ*L), an elevated protein level of 900.0 mg/dL, and a low glucose level of 59 mg/dL (blood glucose, 136 mg/dL). Both blood and CSF cultures were negative, and results of India ink staining of CSF samples were normal. Brain MRI showed no abnormalities in the cerebral parenchyma (Figure 2(a)). An aneurysm (3 mm diameter) was found in the siphon of the right internal carotid artery on MRA (Figure 2(b)).

**Figure 3:**
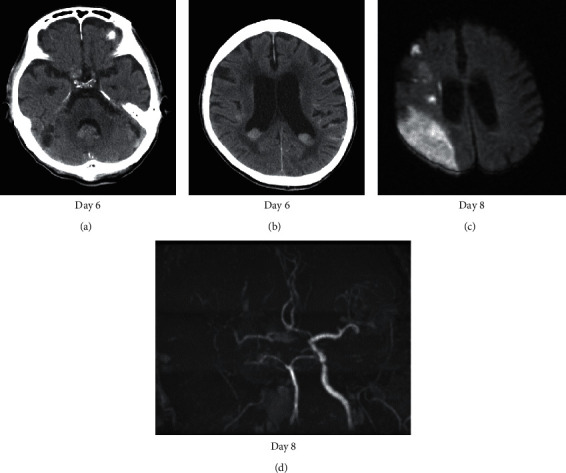
Day 6: head CT showing a subarachnoid hemorrhage (a), (b). Day 8: brain MRI showing a right middle cerebral artery infarction on DWI (c) and MRI showing right internal carotid artery occlusion (d).

**Figure 4:**
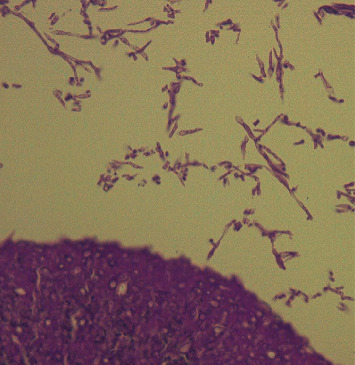
Sinus mucosal biopsy hematoxylin and eosin staining (magnification 400). CT: compound tomography, MRA: magnetic resonance angiography, MRI: magnetic resonance imaging, DWI: diffusion weighted imaging.

Septic meningitis or herpes encephalitis was suspected, and ceftriaxone (2 g/day) and acyclovir (1500 mg/day) were administered. However, serum IgM antibodies to herpes simplex virus (HSV) were negative, and HSV DNA was undetected in the patient's CSF samples. His consciousness deteriorated to E3V1M5 on the sixth day of hospitalization. Repeated head CT revealed a subarachnoid hemorrhage, likely due to the rupture of the aneurysm in the right internal carotid artery (Figures 3(a) and 3(b)). Because treatment with antibiotic and antiviral drugs was ineffective, a fungal infection was suspected. Liposomal amphotericin B (150 mg/day) was administered. On serum examinations, *Cryptococcus neoformans* antigen was negative, *Candida* antigen level was 4 times the normal value (normal range, <2), and *Aspergillus* antigen level was 0.8 ng/mL (normal range, <0.5). Rhinoscopy revealed a fungal mass attached to the middle nasal meatus. Pathological examination of this mass showed numerous spores and mycelium with septae (Figure 4). A culture test characterized the specimen as *Aspergillus*. The patient was diagnosed with invasive *Aspergillus* rhinosinusitis. However, the patient developed left hemiplegia, and his consciousness deteriorated to E1V1M2 on the eighth day of hospitalization. Brain MRI revealed a cerebral infarction in the middle cerebral artery territory and right internal carotid artery occlusion (Figures 3(c) and 3(d)). Although treatment with liposomal amphotericin B was continued, the patient died on the sixteenth day of hospitalization.

## 3. Discussion

The occurrence of meningitis shortly after steroid pulse therapy for visual impairment and the sequential subarachnoid hemorrhage and cerebral infarction, in this case, suggested acute invasive FRS. It is important to consider the possibility of invasive FRS in the differential diagnosis, especially when patients in an immunosuppressed state have meningitis, visual loss, and radiologically proven sinusitis.

*Mucor* and *Aspergillus* are the common causes of acute invasive FRS [5]. When fungal sinusitis is associated with intracranial lesions, it is necessary to consider *Aspergillus* as a causative agent. Pathological confirmation of fungal invasion is necessary to diagnose invasive FRS [5]. When invasive FRS is suspected, a rhinoscopic inspection of the sinuses and mucosal biopsy is necessary [6]. Because different antifungal drugs are used to treat *Mucor* and *Aspergillus* infections, species identification using culture of the biopsied specimen is indispensable. In the case of invasive aspergillosis, the lung is the most frequent site of infection; it rarely occurs only in the sinuses [7]. Immune suppression is a risk factor for acute invasive FRS [4]. However, in this case, the patient was not immunosuppressed. Antinori et al. found that 41 of the 93 patients with *Aspergillus* meningitis were immunosuppressed [8]. In addition, 52 patients of the 93 examined patients did not have a risk for invasive aspergillosis, and 6 of them were suspected to have direct extension of *Aspergillus* from the orbit, ear, or paranasal sinuses to the central nervous system [8]. In this case, we suspected that the Aspergillus spreads from the right maxillary sinus to the right orbital and intracranial regions.

In this case, the patient's *β*-D glucan level was normal. The sensitivity and specificity of the *β*-D glucan test for the detection of invasive fungal infection have been reported to be 63% and 96%, respectively, with a cutoff value of 7 pg/mL [9]. However, in cases of invasive fungal infection, pathological examination and culture are the standard measures for diagnosis, and *β*-D glucan testing has limited utility.

Typical CT findings in FRS are mucus, calcified lesions, and hematomas in the sinuses [10]. On MRI, acute invasive FRS shows high intensity on T2-weighted images, reflecting inflammatory edema and cellular infiltration. In contrast, chronic noninvasive FRS and indolent FRS show low intensity on T2-weighted images [10]. *Aspergillus* rhinosinusitis has marked low intensity on T2-weighted images because of the presence of iron and manganese [11, 12]. In this case, MRI acquired from the previous hospital showed a low-intensity mass in the right maxillary sinus on a T2-weighted image, indicative of *Aspergillus* rhinosinusitis.

In patients with acute invasive FRS due to *Aspergillus*, early recognition and therapeutic intervention are important [13]. Treatments include administration of antifungal drugs and surgical resection or debridement [13]. Amphotericin B and itraconazole have been used for FRS. Recently, voriconazole has been recommended when mucormycosis is excluded and aspergillosis is suspected [13].

Causes of visual impairment in patients with acute invasive FRS involve direct infectious infiltration into the orbit, inflammatory edema, osteomyelitis in the orbit, subperiosteal nodules and abscess, cellulitis in the orbit and face, abscess in the orbit, or thrombosis of the ocular vein [10]. In this case, the cause of visual impairment was unclear in the CT/MRI images. Since no apparent abscess, osteomyelitis, or cellulitis were observed during the clinical course, direct infiltration and inflammatory edema may have possibly caused the visual impairment.

*Aspergillus* is known to invade the vascular wall, causing cerebral infarction and hemorrhage [11]. *Aspergillus* invasion was likely the cause of the sudden aneurysm formation in the right internal carotid artery because prior MRI did not reveal any aneurysms. Direct extension of *Aspergillus* in the right maxillary sinus may have caused infiltration of the vessel walls, leading to the cerebral infarction and subarachnoid hemorrhage.

When sudden visual impairment occurs, and optic neuritis is suspected, steroids are often administered. However, as in the present case, sinusitis due to *Aspergillus* might have been the cause of the patient's manifestations. Therefore, it is necessary to consider all potential causes of visual impairment, especially when cerebrovascular events, such as cerebral infarction, cerebral hemorrhage, or subarachnoid hemorrhage, are involved.
